# Carbonized Lanthanum-Based Metal-Organic Framework with Parallel Arranged Channels for Azo-Dye Adsorption

**DOI:** 10.3390/nano10061053

**Published:** 2020-05-30

**Authors:** Krzysztof Cendrowski, Karolina Opała, Ewa Mijowska

**Affiliations:** Nanomaterials Physicochemistry Department, Faculty of Technology and Chemical Engineering, West Pomeranian University of Technology, Szczecin, Al. Piastow 45, 70-311 Szczecin, Poland; opala.karo@gmail.com (K.O.); emijowska@zut.edu.pl (E.M.)

**Keywords:** anionic dye adsorbent, lanthanum, metal–organic framework (MOF), carbonization

## Abstract

In this contribution, the synthesis of the metal−organic framework (MOF) based on lanthanum that exhibits trigonal prism shape is presented. The length of a single side of this structure ranges from 2 to 10 μm. The carbonized lanthanum-based organic framework (CMOF–La) maintained the original shape. However, the lanthanum oxide was reshaped in the form of rods during the carbonization. It resulted in the creation of parallel arranged channels. The unique structure of the carbonized structure motivated us to reveal its adsorption performance. Therefore, the adsorption kinetics of acid red 18 onto a carbonized metal−organic framework were conducted. Various physicochemical parameters such as initial dye concentration and pH of dye solution were investigated in an adsorption process. The adsorption was found to decrease with an increase in initial dye concentration. In addition, the increase in adsorption capacity was noticed when the solution was changed to basic. Optimal conditions were obtained at a low pH. Kinetic adsorption data were analyzed using the pseudo-first-order kinetic model, the pseudo-second-order kinetic model and the intraparticle diffusion model. The adsorption kinetics were well fitted using a pseudo-second-order kinetic model. It was found that the adsorption of anionic dye onto CMOF–La occurs by hydrophobic interactions between carbonized metal-organic framework and acid red 18.

## 1. Introduction

In the past decade′s amount of colored waste that lands on the groundwaters dangerously grows threatening the natural environment. All over the world researchers are looking for an efficient way to avoid it. Dye wastewaters produced in textile, cosmetic and paint industries are the most difficult to remove from sludges [1]. Many materials were designed and described as an effective adsorbent of dye wastewaters. Azo dyes (e.g., acid red 18) are highly water-soluble dyes among synthetic aromatic dyes and are widely used in industrial applications. The azo dyes represent up to 70% of all dyes used in the production of textiles, study, leather, cosmetics, pharmaceuticals and food [2]. These dyes are composed of -N=N- bond, sulfonic (SO3^−^) groups and complex aromatic molecular structure. The complex aromatic molecular structure is almost non-biodegradable and due to the presence of azo groups shows hepatotoxic and carcinogenic effects in the natural environment [3,4]. For azo dyes neutralization, only adsorption techniques are safe solutions since different photo and electrochemical oxidation techniques generate toxic amine residues in the sludge after their degradation [3]. However, regular consumption of such untreated or poorly treated toxic water shows carcinogenesis in humans [5]. Therefore, dye adsorption from the water solution is a promising way to overcome this problem.

The most popular adsorbent is activated carbon because of physical properties, such as high surface area, bulk density, neutral pH, low ash and low conductivity [6]. However, activated carbon has also many disadvantages such as expensive production, non-selectivity and ineffective in contrary to disperse vat dyes [7]. The others are minerals [8], siliceous materials [9], zeolites [10], metal oxides [11], specific biosorbents [12] and finally metal−organic frameworks (MOFs) [13]. Metal-organic frameworks are crystalline materials with high porosity, structured by various transition metal ions or clusters of metal ions, which occur as nodes in the crystalline framework and bi- or multi-nodal rigid, organic ligands [14]. Except for porosity and high surface area, they are characterized by tunable porosity, ultra-low density, simple synthesis routs and hybrid features [15]. Due to their properties, MOFs found many fields of applications, such as electrochemical application as a material for electrode construction for supercapacitors, modification of electrodes surface [16] or Lithium battery separators [17]. Other common fields of MOF application are gas absorption, drug delivery, selective luminescence sensing [18], catalysis and finally, metals [19] and dye adsorption. Due to the higher porosity and surface area, metal−organic frameworks seem to be a better candidate for adsorption applications, and they become more popular in recent years. However, there is still plenty of room, for new materials based on metal−organic frameworks to discover their variety in terms of morphology and functionality. Therefore, scientists study carbonized metal−organic frameworks in comparison to the activated carbon [20].

The number of publications related to dye adsorption using metal−organic frameworks as an efficient adsorbent is constantly rising every year. Xue-Qing Zhan et al. use the isostructural Zr-based metal−organic framework (UiO-66 and UiO-67) for wastewater treatment. In their study the material was modulated with different pore sizes to enhance adsorption capacity. The researchers customized length of organic ligands using 4,4”-biphenyl dicarboxylic acid and terephthalic acid. Acid Orange 7 (AO7) was used as an example of an azo dye [21]. The amount of the absorbed dye of UiOs was examined and it was 176 and 235 mg g^−1^ for UiO-66 and UiO-67, respectively [22].

Anindita Chakraborty et al. report methyl orange (MO) for adsorptive removal using Cu(BDC) MOF/MgAl-LDH nanocomposite. There the adsorption experiments were conducted at aqueous solution anionic MO dye with different times at a temperature of 298 K. Dye removal of examined MgAl-LDH/Cu-(BDC) metal−organic framework shows efficiency over 99 % after 20 min and reaches adsorption capacity over 600 mg g^−1^ [23]. Xue-Qing Zhan et al. proposed the application of the UiO-66 MOF structure with incorporated iron oxide as an effective magnetic dye adsorbent [24]. Zinc based MOFs showed one of the highest surface areas and are one of the most studied MOF group [25]. Elizabeth Rojas García et al. reported research on iron–benzenetricarboxylate based MOF as an efficient adsorbent of the Orange II [26]. Their studies show that the MOF structure can be successfully used for adsorption of azo dyes.

The lanthanides are a series of 15 elements, from lanthanum (57) to the lutetium (71) often referred to as the 4f block elements [27]. Lanthanides are characterized by high coordination numbers, rich coordination geometries and unique luminescence properties (e.g., long lifetime and narrow emission band) [28,29]. Many publications on synthesis of lanthanide organic frameworks (MOFs-Ln) were reported [30,31]. Until now, only Subbaiah Muthu Prabhu et al. reported the application of MOFs based on lanthanum for Arsenate adsorption. As reported lanthanum-based MOFs shape changes from rod to spheres via replacing of benzoic acid or 1,3,5-benzene tricarboxylic acid with the 1,4-benzenedicarboxylic acid. Due to the lack of the pores, these structures show low surface area 3.50, 12.6 and 5.30 m^2^/g, respectively [32]. Sheta et al. reported lanthanum-based MOFs structures with the shape of nano-rods and nano-sheets using 1, 2-phenylenediamine as an organic linker [33]. In general, lanthanum and other lanthanide-based metal−organic frameworks usually have a shape of the nano-sheets [29] and nano-rods [30].

Dye adsorption on –MOFs-Ln (Ln = Gd, Tb, Eu, Dy) was reported by Cui et al. In their study four organic dyes (cationic methylene blue (MB), anionic methyl orange (MO), anionic orange II (OrII) and neutral methyl red (RD)) were used. The experiment revealed that MOFs-Ln selectively captures methylene blue, while the concentrations of anionic OrII, anionic MO and neutral MR remained unchanged. This class of materials demonstrated adsorption ability only for cationic methylene blue with absorption of 92.6%, 90.8%, 93.8% and 93.3% after 200 min for Tb–MOF, Eu–MOF, Dy–MOF and Gd–MOF, respectively. The amounts of adsorbed MB were calculated for Tb–MOF, Eu–MOF, Dy–MOF and Gd–MOF and it was 147, 141, 133 and 143 mg g^−1^, accordingly [26]. Another report reveals dye adsorption properties of MOFs-Ln (Ln = Eu, Nd, Er, Ce, Dy) on common base dyes: methylene blue, malachite green (MG) and rhodamine B (RhB). The examined materials presented selective adsorption ability to MB and MG dyes and no adsorption of RhB dye. It was established that Eu–MOF, Nd–MOF, Er–MOF, Ce–MOF, Dy–MOF adsorbed 69.21%, 64.59%, 36.40%, 31.38%, 39.70% of MG dye, respectively. The amount of adsorbed MG complex was 1.932 mg g^−1^, 1.803 mg g^−1^ for Eu–MOF, Nd–MOF, accordingly. Likewise, the amount of adsorbed MB complex was calculated for Er–MOF, Ce–MOF, Dy–MOF and was 1.641 mg g^−1^, 1.412 mg g^−1^ and 1.786 mg g^−1^, correspondingly [34]. However, there is no report on MOFs based on carbonized lanthanum for azo-dye adsorption and on their carbonization.

In this contribution, the carbonized lanthanide-based metal−organic framework (CMOF–La) was used as a model azo-dye adsorbent. The MOF structure was used as a substrate for carbon porous material preparation. The carbon structure with parallel arranged channels was formed by carbonization of MOF. Interesting structural properties motivated us to reveal its adsorption potential by using model anionic dye (acid red 18). Presented data clearly indicate that obtained material is highly promising azo-dye adsorbent.

## 2. Results

**Figure 1:**
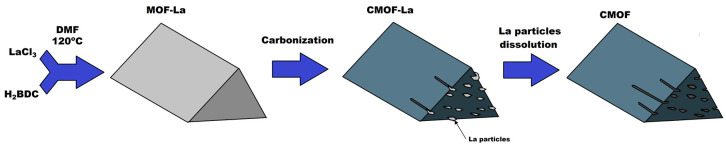
Scheme of lanthanum-based metal–organic framework (MOF-La) and carbonized metal–organic framework before (CMOF-La) and after (CMOF) hydrochloric acid purification.

**Figure 2:**
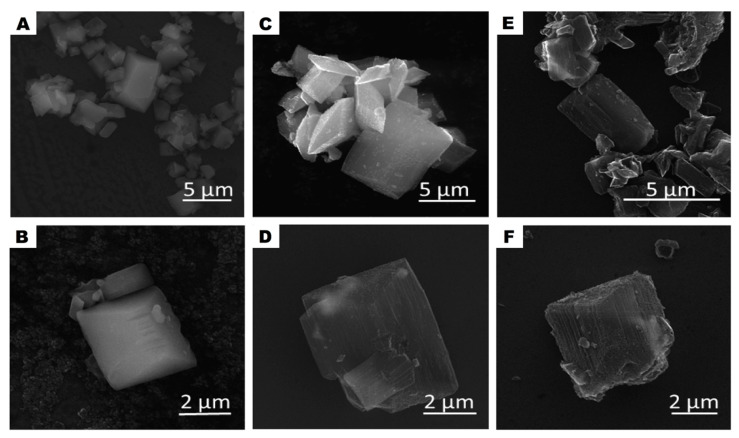
SEM images of MOF-La (**A**,**B**) and MOF-derived carbon structures CMOF-La (**C**,**D**) and CMOF (**E**,**F**).

Schematically, the evolution of the morphology changes of the studied samples is presented in Figure 1 and it is drawn basing on SEM and TEM analysis. SEM images of the pristine MOF sample (A–B), after carbonization (C–D) and acid treatment (E–F) are presented in Figure 2. At each step, the samples (CMOF) exhibited trigonal prism-shaped particles. However, The CMOF–La and acid treated structures are more transparent due to probably more porous structure. In contrary, pristine MOF–La crystals containing metal are not transparent. The scanning electron microscopy images revealed that the surface of the MOF–La crystals is smooth and without any pores. Additional SEM images of CMOF–La-with-La crystals are presented in the supporting information (Figure S1). After CMOF–La hydrochloric acid purification, no La crystals are observed. The SEM images of CMOF structures show channels and pores left after La removal what is more visible in TEM analysis.

**Figure 3:**
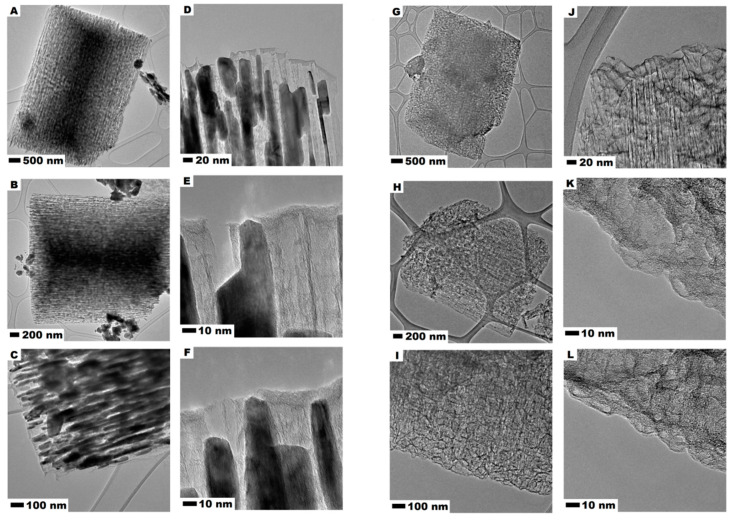
TEM images of MOF-derived carbon CMOF-La (**A**–**F**) and after CMOF (**G**–**L**).

Figure 3 presents TEM images of carbonized CMOF–La (A–F) and acid treated CMOF (G–L) derived carbons. It is observed that the carbonized metal−organic frameworks after carbonization contains plenty of ordered channels which are loaded by metal oxide nanoparticles which form rod-like structures. The metal oxide particles copy the shape of the channels, some of them stick out of the CMOF–La. It is also observed that La-nanoparticles are arranged in one direction. In addition, some La oxide agglomerates can be noticed on the surface of the CMOF–La. The images 3E,F show that formed La oxide nanoparticles are responsible for the CMOF pore structure formation. The CMOF structure with opened channels left after acid treatment in the carbonized MOF is also presented. The comparison of the images in Figure 3A–F, indicates that during the MOF–La carbonization La nanoparticles are extracted from the interior and deposited on the external side of MOF structure. The higher resolution images prove that the channels of the CMOF are open-ended (Figure 3K,L). No crystalline structure was noticed, suggesting the amorphous structure of the obtained carbon nanomaterials.

**Figure 4:**
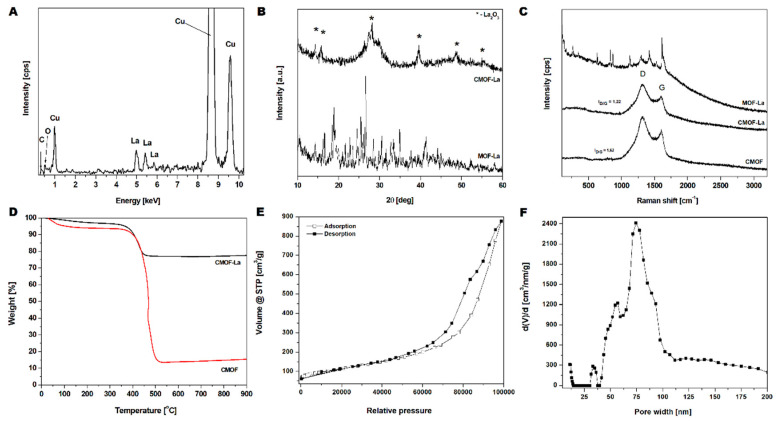
EDS spectrum of the carbonized lanthanum-based MOF (**A**); XRD (**B**) and Raman (**C**) spectra of MOF–La, CMOF–La and CMOF; TGA analysis of CMOF–La and CMOF (**D**); N_2_ adsorption/desorption isotherms (**E**) and pore-width distribution (**F**) of CMOF.

The EDS spectrum (Figure 4A) of CMOF–La structures shown in Figure 4A, clearly demonstrates the presence of carbon, oxygen and lanthanum. Furthermore, CMOF elemental composition, determined by the EDS, proves the successful CMOF purification. The copper signal came from a TEM grid. The crystallographic analysis of pristine MOF–La and obtained derived carbons structures CMOF–La and CMOF were characterized by XRD (Figure 4B). The synthesized MOF–La crystal structure agrees with the experimental data of [La6(BDC)9(DMF)6(H2O)3·3DMF] reported by Yinfeng Han et al [35]. For CMOF–La sample broad peak at around 25° from carbon and sharp peaks at around 4, 5, 28, 39, 48 and 56° from lanthanum oxide were detected. There were no other additional peaks indicating high purity of the obtained materials. Raman spectroscopy was applied in order to determine the structure of the carbonized MOF–La samples. Figure 4C presents the Raman spectra of pristine MOF–La, carbonized sample (CMOF–La) and acid purified material (CMOF). The spectra of the carbonized samples exhibited two typical modes related to the G and D band, which are characteristic for carbon-based structures. The peaks ratio of D to G (ID/IG) increased slightly upon acid purification: from 1.22 to 1.62. This indicates that the acid treatment caused the decrease in structure graphitization of CMOF. The absence of the 2D peak indicates that CMOF–La and CMOF structures were not built from graphene layers [36].

A mass loss in the TGA curves of CMOF–La and CMOF occurred in two stages (Figure 4D). The first mass loss started at 30 °C and corresponded to the removal of adsorbed water and organic solvents (4 wt% and 7 wt% of the samples, respectively).The second mass loss starting at ~370 °C, corresponded to the burring of amorphous carbon material. The carbon nanomaterial burned completely at ~500 °C. The TGA analysis also showed that the content of the metal nanoparticles in the carbonized and purified CMOF was ~63 wt% lower than that in CMOF–La sample. The BET surface measurements and histogram of pore-width distribution of the CMOF are shown in Figure 4E,F, respectively. The nitrogen adsorption/desorption isotherms of carbonized samples were similar to type IV isotherms. The hysteresis loop can be classified as mixed H3 and H4 type of hysteresis loops. The specific surface area reached 408 m^2^/g with a pore volume of 1.3 cm^3^/g and an average pore radius of 84.2 nm (Figure 4F). The average pore radius according to the TEM images analysis was about 13 nm, which corresponds to the size of the La rods (supplementary Figure S2).

**Figure 5:**
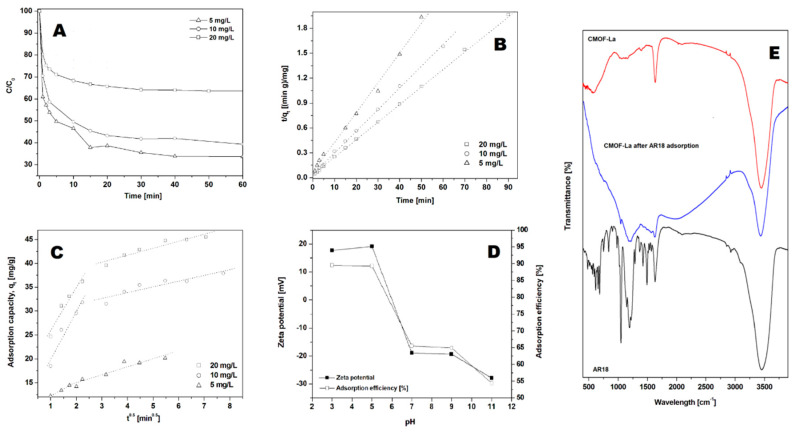
Effect of initial dye solution concentration (**A**) on adsorption capacity of the acid red 18 (AR18) onto CMOF. Pseudo-second-order kinetics (**B**) and intraparticle diffusion model (**C**) of acid red 18 adsorptions onto CMOF. Influence of initial pH and Zeta potential on dye adsorption (**D**). Experimental conditions: T = 20 °C, pH = 7. FT-IR spectra of carbonized MOF, CMOF after adsorption and AR18 (**E**).

Acid red 18 (AR18) adsorption experiments were performed on CMOF with the constant adsorbent amount of 40 mg/L (Figure 5A) and various initial dye concentrations at pH of 7 at 20 °C. As the amount of AR18 varies from 5 to 20 mg/L, the efficiency of adsorption of acid red 18 was 67 % for 5 mg/L, 58 % for 10 mg/L and 37 % for 20 mg/L (after 60 min), respectively. In all studied experiments, acid red 18 adsorption proceeded in two stages. In the first stage adsorption capacity of AR18 increased rapidly up to 5 min and thereafter it proceeded at a slower rate until equilibrium was attained. The time necessary to reach the equilibrium point increases with the increase in CMOF to the AR18 ratio. The highest acid red adsorption capacity (47.35 mg/g) was observed for the dye:adsorbent ratio equal 1:8 (Table 1).

The highest correlation coefficients were observed for the pseudo-second-order kinetic model (Figure 5B). The correlation coefficients for the intraparticle diffusion kinetic model were lower than for the calculated pseudo-second-order kinetic model (see Figure 5C and Table 1). This suggests that the pseudo-second-order adsorption mechanism is predominant.

The effect of the initial pH on the adsorption efficiency at equilibrium of acid red 18 is shown in Figure 5D. The adsorption efficiency at equilibrium of AR18 increases significantly with a decrease in the initial pH. The highest adsorption efficiency was observed at pH 3. When the pH was increased to 7 and higher, the adsorption efficiency decreased to 65–55 %. The effect of pH has an important influence on controlling the adsorption process. Analysis of the zeta potential of the absorbent showed that with an increase in pH the surface charge changes from the positive value to the negative, reaching zero net charges at pH = 6. This can be explained by dissociation of AR18 into sodium cations and sulfur anions in acid aqueous solution. Positive charges at the surface of CMOF (in pH below 6.5) enhance the adsorption of anionic dye through the electrostatic interaction. Figure 5D shows strong correlation between zeta potential and adsorption capacity.

The FT-IR spectra of CMOF–La before and after dye adsorption in pH range from 3 to 11 are presented in Figure 4E. FTIR analysis was carried out to examine the interaction between the dye and carbonized metal−organic framework. The FTIR spectra of acid red 18 and MOF–La samples were measured at a range of ~400–4000 cm^−1^. The bands at around 3454, 3463, 3434 and 3487 cm^−1^ correspond to the -OH stretch at all of the samples. Acid red spectrum shows peaks originating from C–C vibrations at around: 1039, 1292, 1421, 1493 and 1635 cm^−1^. The bands at ~483– 691 and 1365 cm^−1^ correspond to the C-S stretch. CMOF spectrum demonstrates the band at ~1631 cm^−1^ which can be attributed to C=C vibrations. The band of ~1625 cm^−1^ of CMOF after adsorption was assigned to the aryl-substituted C=C stretching; the ~1203 cm^−1^ corresponds to the aromatic C-H bond. The peaks from the C-H vibrations and C=C stretching can be attributed to the AR18 presence. The FT-IR analysis indicated the presence of AR18 in the sample after adsorption but did not show any characteristic peaks originating from chemical bonding between dye molecules and the adsorbent.

## 3. Discussion

The possible mechanism of adsorption on carbon materials are the π-π interactions, hydrogen bonding and Van der Waals interactions [37,38]. The π-π interactions are independent on the influence of environmental factors such as pH, ionic concentration, temperature and concentration [38]. The reports on AR18 adsorption onto carbon nanostructures show that π-π interactions are the dominant mechanism [39,40,41]. Taking into account the changes in the functional groups in the CMOF before and after dye adsorption, electrostatic interactions seems to be the dominant mechanism for the adsorption of acid red 18 onto carbonized MOF. The adsorption capacities are dependent on pH, suggesting the physical adsorption through the hydrophobic effects between CMOF and AR18 [42].

Azo dyes (e.g., acid red 18) are one of the synthetic dyes that are used in many industries. The treatment and disposal of dye-contaminated wastewater are one of the most serious environmental problems. Intermediate products of azo dyes are toxic, mutagenic and carcinogenic to humans and aquatic life. For this reason, there are many reports on the adsorption processes of this group of dyes. Table 2 lists the comparison of parameters and adsorption capacity of AR 18 dye onto carbonized MOF structures and other carbon adsorbents. The maximum adsorption capacity Qe was 44.26 mg/g onto CoOF, similar to the CMOF. In accordance with the state of art on acid red 18 adsorption, the highest maximum adsorption capacity was observed for chitosan-based samples (691 mg/g) [4]. According to the W.H. Cheung et al. the adsorption of the acid dyes on the chitosan occurs through the electrostatic attraction between two counterions: the amino groups of chitosan and sulfate groups of acid dye [43]. As reported by Y.C. Wong et al. the adsorption efficiency may mainly be attributed to the chemical structure and molecular size. Since larger dye ions, like AR 18, do not completely penetrate the adsorbent pore and preferentially adsorb on the outer surface [2]. The summarized data (Table 2) show that carbonized MOF–La shows high adsorption capacity. This can be due to the wide pores formed during MOF–La carbonization. Lanthanum oxide shows low toxic effects [44,45] and their toxic effect increases when the size decreases from micro to nanoscale [46]. Due to the fact that the material contains a low amount of metal oxide residues, its toxicity can be comparable to amorphous carbon structures. The adsorption acid red 18 onto CMOF is determined by pseudo-second-order similar to the other carbon structures listed in Table 2.

## 4. Materials and Methods

### 4.1. Materials

Lanthanum (III) chloride heptahydrate (LaCl_3_·H_2_O), terephthalic acid (H_2_BDC) were purchased from Sigma Aldrich (MERCK, Darmstadt, Germany). N,N-dimethylformamide was acquire from Honeywell (Warsaw, Poland). Thirty-five percent hydrochloric acid (HCl) was bought from Chempur (Piekary Slaskie, Poland).

### 4.2. MOF–La Synthesis

The MOF–La was obtained via a solvothermal route. A mixture of lanthanum (III) chloride (655 mg) and terephthalic acid (198 mg) was dissolved in 240 mL N, N-dimethylformamide solution. The prepared mixture was then stirred for 24 h at a temperature of 110 °C. After 24 h obtained composition was cooled to the room temperature and centrifuged to remove the solvent.

### 4.3. Carbonization of MOF–La

Porous carbon derived from MOF–La was obtained by direct thermolysis of the as-prepared sample. The sample was introduced to a silica quartz boat and then inserted into the center of a tubular furnace. First, the furnace was heated from room temperature to 115 °C in 1 h to remove the solvent, then heated to the temperature of 1000 °C. The black MOF–La powder was maintained at this temperature for 3 h with an Ar flow of 100 sccm. After the carbonization process, the tubular furnace was cooled to room temperature. The materials referes to CMOF-La.

Next, the material was purified to remove metal from the structure. Therefore, the carbonized sample was dissolved in 10 mL of 35% HCl and sonicated for 1 h. Then the solution was centrifuged, and black powder was collected. This sample is designated as CMOF.

### 4.4. Dye Adsorption

Dye adsorption experiments were carried out in sealed Erlenmeyer flask, where 250 mL of dye solution with known initial dye concentration was placed. The initial concentrations of dyes were varied from 5 to 20 mg of Acid red 18. The adsorbent concentration was constant at 40 mg/L. The flask with a dye solution was agitated with a magnetic stirrer in order to achieve homogeneity. To observe the effect of pH in the process the experiments were carried out at different pH values from 3 to 11 of the dye solutions with the dye and adsorbent concentration 10 mg/L and 40 mg/L, at room temperature. The pH was adjusted by adding a few drops of diluted hydrochloric acid (0.1 N HCl) or sodium hydroxide (0.1 N NaOH). During adsorption 4 mL of aqueous sample was taken from the solution and the liquid was separated from the adsorbent by centrifugation at 6000 rpm for 5 min. The dye concentration was determined using a spectrophotometer measuring maximum absorbance at 509 nm. The equations used for calculation pseudo-first and second-order kinetic models are presented in the supplementary information.

### 4.5. Characterization Techniques

The morphology of the samples was characterized with a scanning electron microscope (VEGA3 TESCAN, Brno, Czech Republic) and transmission electron microscope (Tecnai G2 F20 S-TWIN, FEI). X-ray diffraction (XRD) patterns were carried out using and X’Pert Philips Diffractometer (X’Pert PRO Philips diffractometer, Almelo, Holland) with a Cu anode (Kα1 = 1.54056 Å) to examine the crystal composition of the samples. The Raman spectra were obtained by Renishaw microscope (λ = 785 nm). Thermogravimetric analysis (TGA) was conducted under an argon flow with heating rate of 10 °C min^−1^, using TA Instrument SDT Q600 (TA Instrument, New Castle, DE, USA). N2 adsorption/desorption isotherms were obtained using a QuadraSorb SI (Quantachrome Instruments, Boynton Beach, FL, USA). Specific surface area was calculated according to the Brunauer–Emmett–Teller (BET) method. FT-IR transmittance spectra were recorded on Nicolet 6700 FT-IR Spectrometer (Thermo Fisher Scientific, Waltham, MA, USA).

## 5. Conclusions

In conclusion, carbon trigonal prism structure was obtained by the carbonization of La-based MOFs at high temperatures under an inert gas atmosphere. During MOF carbonization, lanthanum formed oxide crystals in the shape of rods. The crystals are formed and extracted during carbonization in one and the same direction from the carbonized MOF. The obtained carbonized organic frameworks were composed of amorphous carbon, with paralleled arrange channels formed upon the extraction of lanthanum oxide.

In this study, we investigated the potential of application carbonized MOF structure based on lanthanum for adsorption of commercial dyes (acid red 18). The kinetic study was performed based on pseudo-first-order, pseudo-second-order and intraparticle diffusion kinetic models. The data indicate that the adsorption kinetics follow the pseudo-second-order kinetic model. The results showed that the adsorption capacity of AR18 onto CMOF increased with the decrease in the initial pH. The adsorption efficiency is strictly related to the changes in the zeta potential.

The proposed MOF structure shows potential in the application for azo-dyes removal exhibiting high adsorption capacity. Therefore, channels formed via the extraction of lanthanum oxide particles can serve for molecules adsorption and storage.

## Figures and Tables

**Table 1:** Parameters for the adsorption of acid red 18 onto acid treated carbonized La based metal-organic frameworks (CMOF).

C_0_ (mg/L)	Q_e,_ Exp (mg/g)	Pseudo-First-Order			Pseudo-Second-Order			Intraparticle Diffusion Model		
		k_1_ (min^−1^)	q_e_ (mg/g)	R^2^	k_2_ (g/mg·min)	q_e_ (mg/g)	R^2^	K_p_ (mg/g min^0.5^)	C (mg/g)	R^2^
5	20.93	0.1365	24.34	0.9400	0.0122	27.86	0.9994	2.99	9.21	0.996
10	38.28	0.3777	29.39	0.9400	0.0183	38.27	0.9994	10.06	9.75	0.9363
20	47.32	0.4609	35.02	0.9450	0.0194	47.35	0.9944	9.01	16.88	0.9356

**Table 2:** Comparison of acid red 18 adsorption.

Adsorbent Type	Adsorbent [mg]	C_0_ [mg/L]	Volume [mL]	Temperature [°C]	Time [min]	pH	Q_e_ [mg/g]	Kinetic Model (R^2^)	Ref.
Carbonized Phragmites australis	400	40–140	50	25	450	6.6	80.94	Elovich (0.942)	[39]
polypyrrole-chitosan composites	50	50	100	26	60	7.0	100.00	Second-order (1.0000)	[40]
Activated carbon	1000	300	N/A	30	240	7.0	29.30	Second-order (0.998)	[42]
Chitosan/Carbon Nanotube	50	300	20	29	120	7.0	691.0	Second-order (0.985)	[4]
Ordered mesoporous TiO2/activated carbon	150	80	250	25	30	7.0	138.9	Second-order (0.996)	[47]
Semi-IPN hydrogel composites	60	200	100	25	300	2.0	345.7	Second-order (0.9996)	[48]
Activated carbon	25	1000	25	25	150	7.0	599.821	Second-order (0.987)	[49]
Carbonized cobalt based organic framework-CoOF	10	100	10	22	90	7.0	43.309	Second-order (1.000)	[50]
CMOF	40	10	250	25	30	7.0	38.27	Second-order (0.9993)	This work
CMOF	40	20	250	25	30	7.0	47.35	Second-order (0.9994)	This work

## Supplementary Materials

The following are available online at https://www.mdpi.com/2079-4991/10/6/1053/s1, Figure S1: SEM images of carbonized lanthanum-based MOF, Figure S2: Size distribution of the La-nanoflakes.
