# Supplementary material for: Carbonized Lanthanum-Based Metal-Organic Framework with Parallel Arranged Channels for Azo-Dye Adsorption
Source: Nanomaterials (Basel). 2020 May 30;10(6):1053. doi: 10.3390/nano10061053 (PMC7353049; doi:10.3390/nano10061053)
Supplement: Supplementary file 1 [file nanomaterials-10-01053-s001.pdf]

nanomaterials

Supplementary Materials

Carbonized Lanthanum-Based Metal-Organic Framework with Parallel Arranged Channels for Azo-Dye Adsorption

Krzysztof Cendrowski *, Karolina Opała and Ewa Mijowska

Nanomaterials Physicochemistry Department, Faculty of Technology and Chemical Engineering, West Pomeranian University of Technology, Szczecin, Al. Piastow 45, 70-311 Szczecin, Poland; opala.karo@gmail.com (K.O.); emijowska@zut.edu.pl (E.M.)

* Correspondence: kcendrowski@zut.edu.pl; Tel.: +48-91-4496033

Pseudo-First-Order and Pseudo-Second-Order Kinetic Model

The pseudo-first-order kinetic model (1) in his linear form (2) was calculated according to following equation:

$$\frac{dq}{dt} = k_1(q_e - q_t) \tag{1}$$

$$\ln(q_e - q_r) = \ln q_e - k_1 t \tag{2}$$

In the presented equation, following values represents: k_1 (min^{-1}) - first-order rate constant adsorption; q_e and q_t - adsorption capacity; and t is the selected time.

The pseudo-first-order kinetic model (1) in his linear form (2) was calculated according to following equation:

The pseudo-second-order kinetic model was expressed by following equation (3):

$$\frac{dq}{dt} = k_2(q_e - q_t)^2 \tag{3}$$

The linear form of pseudo-second-order kinetic model equation (4):

$$\frac{t}{q_t} = \frac{1}{k_2 q_e^2} + \frac{1}{q_e} t \tag{4}$$

In the presented equation, following values represents: k_2 ($g\ mg^{-1}\ min^{-1}$) - rate constant for the pseudo-second-order adsorption kinetics; q_e and q_t - adsorption capacities; and t is the selected time.

The results obtained from the pseudo-first-order and the pseudo-second-order kinetic models together with the corresponding correlation coefficients R^2 values are given in Table 1. According to the correlation coefficient, the adsorption of Acid Red 18 is described by the pseudo-second-order kinetic model . The correlation coefficient in this model (R^2>0.99 for all studied concentrations) was

higher than the one counted for the pseudo-first order (R^2= 0,98 ÷ 0,84). Differences in values between experimental and calculated *qt* for the pseudo-second-order kinetic model are insignificant for all studied concentrations. The equations used to evaluate these models are presented in the supporting information.

Intraparticle Diffusion Model

The intraparticle diffusion model, was used in the following equation, proposed by the Webber and Morris [1] (5):

[illegible] (5)

In the presented equation, following values represents: C (mg/g) is the intercept and k_p (mg/g · min) is the intraparticle diffusion rate constant. The intraparticle diffusion kinetic model ($R^2 \leqslant 0.828$) is lower than calculated pseudo-second-order kinetic model.

The regression was linear but did not pass through the origin (see Figure S2). Since in the Figure S2 are presented two linear sections with different slopes, the interparticel diffusion in not independently controlling rate. It means that the diffusion of dye onto CoOF occurs in two stages. The first and faster linear section (dashed line) is equivalent for the boundary diffusion effect. The second section (solid line) was attributed to the intraparticle diffusion and limits step of adsorption. These two phases suggested that the adsorption process had proceeded by the surface adsorption and the intraparticle diffusion. Wang et al. (Wang at al. 2007) and Dogan et al. (Dogan et al. 2009) had similar observations. In the beginning part of the plot indicated the boundary layer effect. In the second linear part of the plot was caused by the diffusion of intraparticle or pore.

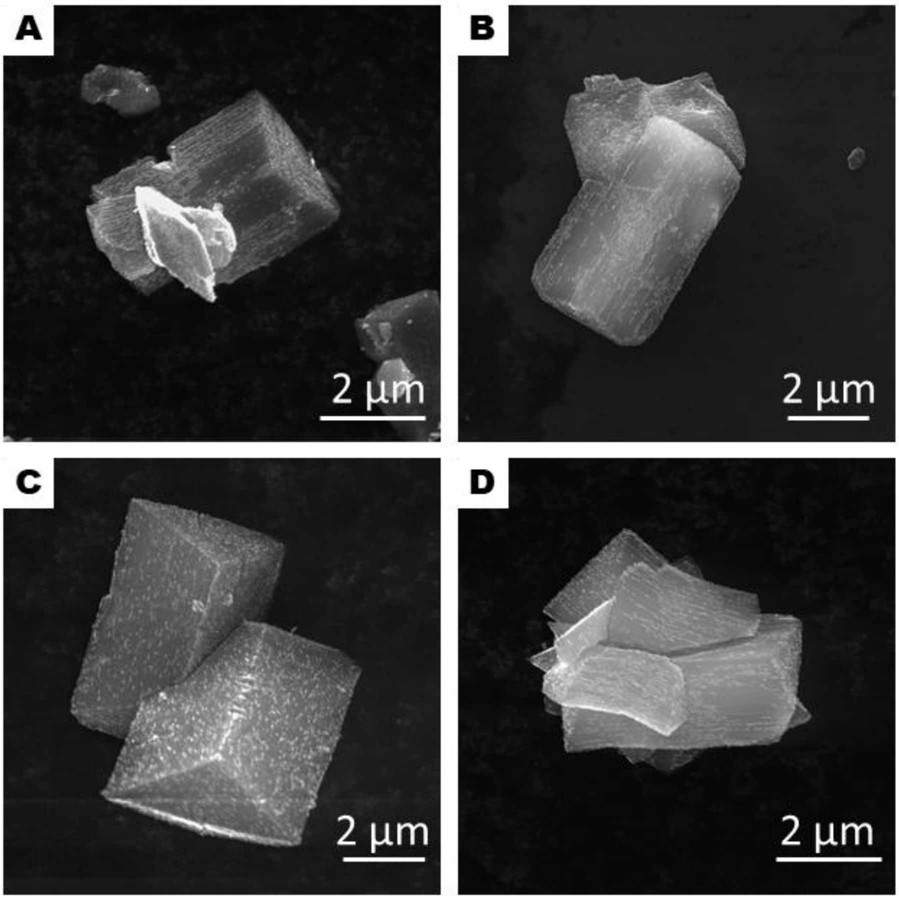

Figure S1. SEM images of carbonized lanthanum based MOF.

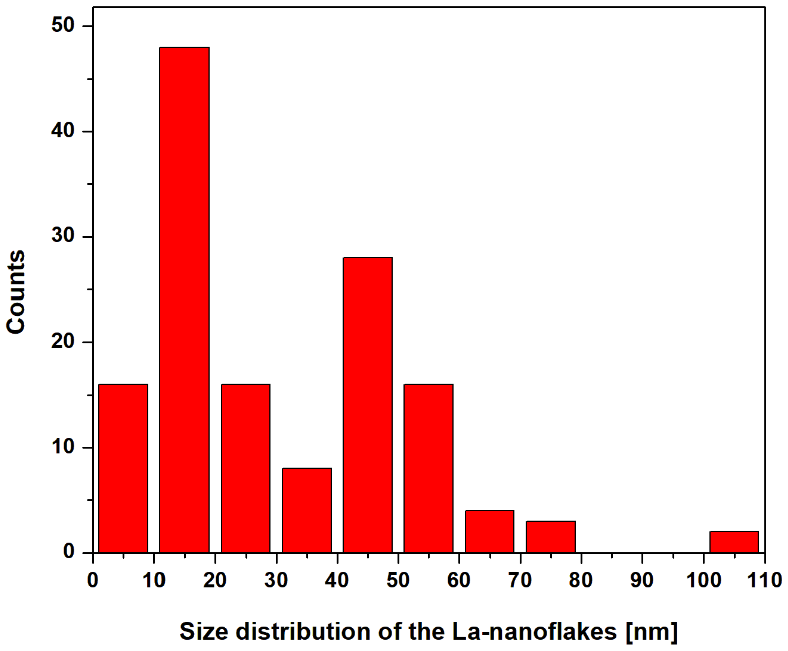

Figure S2. Size distribution of the La-nanoflakes.

Reference:

[1] Weber, W.J.; Morris, J.C. Kinetics of adsorption on carbon from solution. *J. Sanit. Eng. Div* **1963**, *89*, 31–60
